# Quantitative Determination of Chlormequat Chloride Residue in Wheat Using Surface-Enhanced Raman Spectroscopy

**DOI:** 10.1155/2018/6146489

**Published:** 2018-07-10

**Authors:** Shizhuang Weng, Mengqing Qiu, Ronglu Dong, Fang Wang, Jinling Zhao, Linsheng Huang, Dongyan Zhang

**Affiliations:** ^1^Anhui Engineering Laboratory of Agro-Ecological Big Data, Anhui University, 111 Jiulong Road, Hefei 230601, China; ^2^Science and Technology on Communication Networks Laboratory, Shijiazhuang 050000, China; ^3^Hefei Institute of Physical Science, Chinese Academy of Sciences, 350 Shushanhu Road, Hefei 230031, China

## Abstract

A simple and sensitive method for detection of chlormequat chloride residue in wheat was developed using surface-enhanced Raman spectroscopy (SERS) coupled with chemometric methods on a portable Raman spectrometer. Pretreatment of wheat samples was performed using a two-step extraction procedure. Effective and uniform active substrate (gold nanorods) was prepared and mixed with the sample extraction solution for SERS measurement. The limit of detection for chlormequat chloride in wheat extracting solutions and wheat samples was 0.25 mg/L and 0.25 *μ*g/g, which was far below the maximum residual value in wheat of China. Then, support vector regression (SVR) and kernel principal component analysis (KPCA), multiple linear regression, and partial least squares regression were employed to develop the regression models for quantitative analysis of chlormequat chloride residue with spectra around the characteristic peaks at 666, 713, and 853 cm^−1^. As for the residue in wheat, the predicted recovery of established optimal model was in the range of 94.7% to 104.6%, and the standard deviation was about 0.007 mg/L to 0.066 mg/L. The results demonstrated that SERS, SVR, and KPCA can provide the accurate and quantitative determination for chlormequat chloride residue in wheat.

## 1. Introduction

Plant growth regulator can increase crop production, improve quality, and enhance stress resistance [1, 2] through regulating cell growth, cell division, rhizogenesis, germination, blossom and maturation. Chlormequat chloride [3, 4] is an excellent plant growth regulator which is widely applied in wheat, rice, cotton, tobacco, corn, and tomato. Taking advantage of inhibition effect of cell elongation, chlormequat chloride improves the resistance of crops to drought, waterlogging, saline-alkali soil, or lodging. However, the residue in agricultural grains induced by the unreasonable and excessive application is health hazard for humans and animals, leading to economic losses to agricultural trade [5–7].

Chlormequat chloride detection methods as gas [8] or liquid chromatography [9, 10] coupled with mass spectrometry are highly selective and very accurate; thus, they are considered as standard detection methods. Nevertheless, these methods are not practical and convenient due to expensive and toxic reagent consumption, high analysis complexity, and requirement of large laboratory instruments and trained personnel [11, 12]. Meanwhile, considering the large-scale residue, the detection process must be simple and rapid. Therefore, a method employing portable equipment and simple detection procedure would be more suitable for detection of chlormequat chloride in grains.

Spectroscopic methods are promising tools for detection of farm chemical residue because they are simple, rapid, specific, and partially or completely automatic. The commonly used spectroscopic methods include near-infrared spectroscopy (NIR) [13, 14], Fourier-transform infrared spectroscopy (FTIR) [15, 16], Raman spectroscopy (RS) [17, 18], and surface-enhanced Raman spectroscopy (SERS) [19, 20]. NIR and FTIR are unsuitable for residue detection in grains because of severe interference from aqueous environments. RS can provide comprehensive and fingerprint information of analyte without impact from aqueous phase, but its application is also limited by the low sensitivity for small cross-section of Raman scattering. By contrast, SERS is the most promising technique in trace detection because it greatly improved the sensitivity of RS through large enhancement of inelastic Raman scattering of molecule absorbed to the surface of nanoscale noble metal like silver, gold, and copper [11, 21]. Meanwhile, SERS inherits the advantage of RS with rapidity to provide fingerprint and comprehensive information. Due to these advantages, SERS is broadly applied in detection and discrimination of farm chemicals [22, 23], toxins [24], additives [25], drugs [26], and biomacromolecules [27]. Particularly, for farm chemicals, SERS has been used to detect isofenphos-methyl [12], chlorpyrifos [28], thiram [23], fenthion [29], triazophos [30], and so on. In addition, the intelligent analysis of spectra using chemometric methods can initiate acquisition of analyte information independent of professionals, which makes SERS easy and simple for popularization and application in detection. Polynomial fitting [31], derivative transformation [12], and asymmetric least squares [32] are often used to deduct baseline shift caused by the fluorescent background and other interference effects. Principal component analysis [12] and nonnegative factorization [33] are commonly adopted for extracting main information and reducing data dimensions. Some other methods such as multiple linear regression (MLR) [34], partial least squares regression (PLSR) [12], artificial neural network [35], and support vector regression (SVR) [36] are usually employed to develop the regression models for quantitative determination of substances with excellent predictability.

The objective of this study is to develop a simple and sensitive SERS method for quantitative determination of chlormequat chloride in wheat coupled with some chemometric methods on a portable Raman spectrometer, in which pretreatment of wheat samples is performed using a two-step extraction procedure. To the best of our knowledge, this paper is the first to report detection chlormequat chloride in grains using SERS technique.

## 2. Materials and Methods

### 2.1. Materials

Wheat samples were purchased from Hefei Zhougudui market. Chlormequat chloride powder (99.6%) was obtained from Beijing Puxi Technology Co., Ltd. Anhydrous methanol was acquired from Sinopharm Chemical Reagent Co., Ltd. Cetyltrimethylammonium bromide (CTAB), hydrogen tetrachloroaurate, trisodium citrate, L-ascorbic acid, sodium borohydride, and silver nitrite were purchased from Aladdin Industrial Corporation.

### 2.2. Sample Preparation

The pretreatment method for wheat was developed based on the extraction method in gas chromatography (GC). Wheat was first grinded using a pulverizer (Xinrui DFT-150, Changzhou, China) and filtered through 10-mesh sieves. Wheat powder of 5.00g was added with 15 mL of methanol in 50 mL centrifuge tube and then vibrated for 10 min. The mixture was centrifuged at 4000 rpm for 3 min, and the supernatant was moved to the concentrated bottle. Wheat residue was extracted using 10 mL of methanol again, and the supernatant was also moved to the concentrated bottle. The supernatant was evaporated to dry on a Rotavapor (Yarong RE-52A, Shanghai, China) and redissolved in 5 ml of methanol.

Wheat extracting solutions containing different chlormequat chloride were then prepared. The obtained extraction solution was used to dissolve chlormequat chloride powder for getting the solution of 20, 10, 5, 2.5, 1, 0.5, and 0.25 mg/L. Additionally, to simulate actual residue, wheat powder was spiked with chlormequat chloride to yield final residue at 10, 5, 2.5, 1, 0.5, and 0.25 *μ*g/g. The contaminated samples were extracted using the above pretreatment method.

### 2.3. SERS Measurement

The synthesis of gold nanorods (GNRs) was performed using a seed-mediated growth method previously developed by El-Sayed [37]. GNRs sol-solution was centrifuged at 8000 rpm for 10 min to get gray colloid, and 2 *μ*L of GNRs colloid was dropped on silicon chip. After the droplet became dry, 2 *μ*L of testing solution was dropped on the GNRs film. When the solvent was evaporated to dry, spectra were collected on a portable Raman spectrometer (B&WTEK, i-Raman785® Plus, USA) equipped with a 785 nm laser of 150 mW. The measurement was performed with 3 scans and exposure time of 5 s, and the spectral resolution was 2 cm^−1^ in the Raman shift range of 600 cm^−1^ to 1800 cm^−1^. The spectra of 10 from five different spots on each sample were collected as the representative spectra. Five samples were measured for chlormequat chloride residue of each concentration.

**Figure 1:**
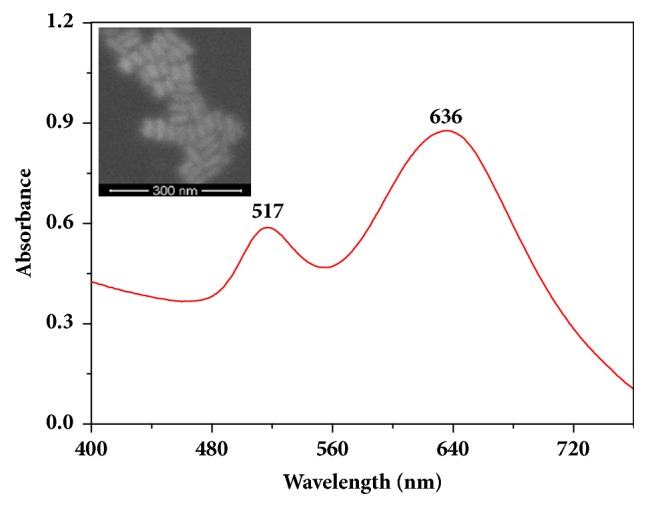
Ultraviolet-visible absorption spectrum of the prepared GNRs colloid; the inset is SEM image of GNRs.

Absorption spectra of the GNRs were recorded on an ultraviolet-visible (UV–Vis) spectrometer (UV-2600, Shimadzu, Japan). Morphologies of the GNRs were surveyed using the scanning electron microscope (SEM) image on a JSM 7500F microscope (JEOL Ltd., Tokyo, Japan). As shown in Figure 1, the GNRs exhibited two plasmon resonance bands of 517 and 636 nm which correspond to electron oscillations along the short and long axes of nanorods. SEM images revealed that the GNRs are ordered and uniform.

### 2.4. Chemometric Methods

The obtained spectra were first baseline-corrected using asymmetric least squares method to eliminate baseline and linear slope effects [32]. Subsequently, kernel principal component analysis (KPCA) was applied for the feature extraction of spectral data to obtain main information and reduce the dimension. KPCA is a nonlinear PCA developed with the kernel method. Concretely, KPCA projects spectra to the high-dimensional space and achieves separable data. Then, the obtained high-dimensional data is transformed into many principal variables (scores) using principle of principal component analysis. These variables are used to describe and replace the original spectra with advantage of weakening noise interference [38]. Radial basis function (RBF) was selected as the kernel function in KPCA for its high effectiveness in training process, and the effect of different width of kernel function (*σ*) on feature extraction was discussed.

To examine accurate and quantitative determination of analyte further, MLR, PLSR, and SVR were used to develop the regression models. MLR is a regression algorithm that is very efficient in building calibration models when the number of samples is more than that of variables. PLSR is one of the most robust and reliable tools in the development of a multivariate calibration model. Based on the linear algorithm, PLSR is often applied to predict a set of dependent variables from a large set of independent variables. PLSR decomposes the spectral array and concentration array with considering their relationships. Corresponding calculation relationships are strengthened for the better correction model. SVR is a variation of support vector machine with introduction of insensitive loss function. Despite finite sample, SVR still possesses excellent robustness and high sensitivity through balancing complexity and learning ability of model. Meanwhile, with the aid of kernel function, SVR can project data into the high-dimensional space for obtaining higher analysis accuracy. Moreover, RBF was also selected as the kernel function of SVR. Considering the performance of obtained regression models highly depends on penalty coefficient (C) in loss function and width of kernel function (*σ*), the optimal values of C and *σ* are obtained by traversing their empirical values [36]. The performances of the above models were quantitatively evaluated using 5-fold cross-validation method with root mean square error of cross-validation (RMSECV). All data analyses and validation of chemometric methods were performed in MATLAB 2013a (MathWorks Inc., Natick, MA, USA).

## 3. Results and Discussion

### 3.1. SERS of Chlormequat Chloride

**Figure 2:**
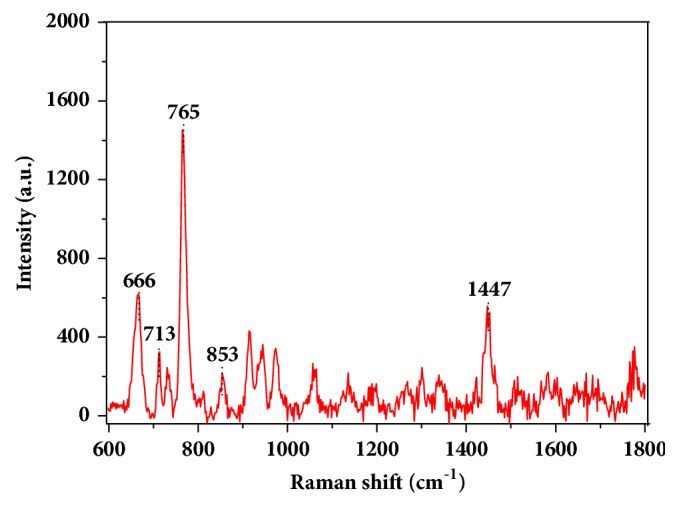
Raman spectra of pure chlormequat chloride powder.

The characteristic peaks reflect the information of molecular vibration and rotation, and these peaks are the basis for analysis and detection of substance using Raman or SERS technique. To determine the characteristic peaks of chlormequat chloride, pure chlormequat chloride powder was placed on the silicon wafer, and then Raman spectra were obtained through direct laser irradiation on it. The main Raman peaks of chlormequat chloride at 666, 713, 765, 853, and 1447 cm^−1^ were observed in Figure 2. According to relevant Raman peak assignment and structure of chlormequat chloride molecule, the peaks at 666 and 765 cm^−1^ were attributed to C-Cl stretching vibration in synclinal and synperiplanar conformation. The bands at 713 and 1447 cm^−1^ can be associated with CH_2_ oscillating vibration and CH_2_-Cl bending vibration (in plane), respectively. Furthermore, the peak at 853 cm^−1^ was assigned to C-N symmetric stretching vibration.

**Figure 3:**
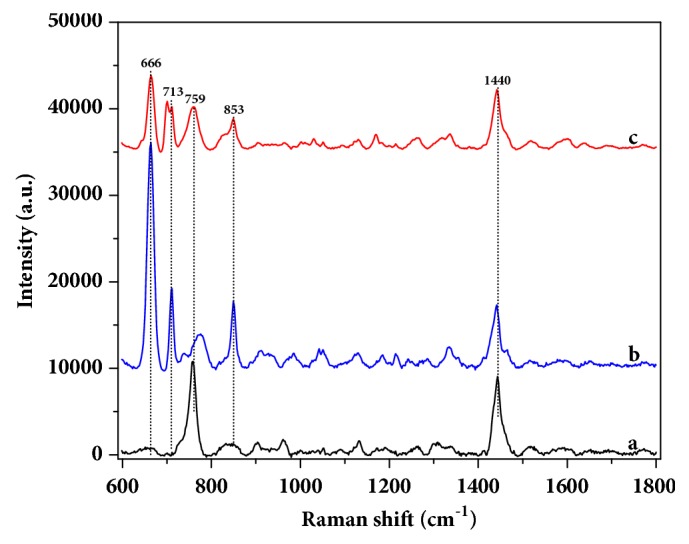
SERS spectra of gold nanorods (a), 100 mg/L of chlormequat chloride in methanol (b), and 20 mg/L of chlormequat chloride in wheat extraction solution (c).

However, the characteristic bands of SERS of molecule in complex media may have changes, which is mainly due to influence of Raman active substrate and background signals of complex media. Then, SERS spectra of GNRs, 100 mg/L of chlormequat chloride in methanol, and 20 mg/L of chlormequat chloride in wheat extraction solution were measured and shown in Figure 3. As shown in figure, the bands at 759 and 1440 cm^−1^ were for SERS of GNRs, which were attributed to CTAB residue. Furthermore, the two bands influenced the appearance of peaks at 765 and 1447 cm^−1^ of chlormequat chloride. Conversely, the other peaks at 666, 713, and 853 cm^−1^ were not affected by the substrate and complex media. Therefore, the peaks at 666, 713, and 853 cm^−1^ were the key features for detection of chlormequat chloride residue in wheat extraction solution using SERS.

**Figure 4:**
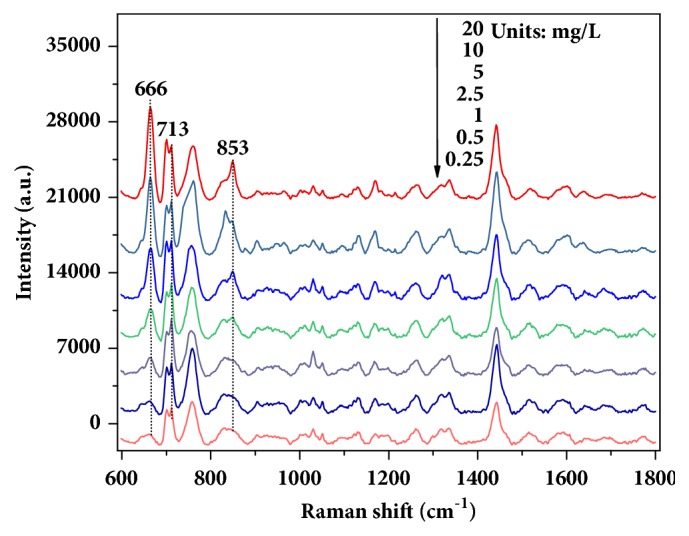
Spectra of 20, 10, 5, 2.5, 1, 0.5, or 0.25 mg/L of chlormequat chloride in wheat extraction solution.

Then, SERS spectra of wheat extraction solution with 20, 10, 5, 2.5, 1, 0.5, or 0.25 mg/L of chlormequat chloride were measured with the uniform GNRs (Figure 4). As seen in the figure, the intensity of characteristic peaks weakened concomitantly with the decrease in concentration, which suggests SERS have potential for quantitative analysis. When the concentration of chlormequat chloride in solution was 0.25 mg/L, the peak at 713 cm^−1^ was still obvious, but the peaks at 666 and 853 cm^−1^ were just dimly visible. The phenomenon indicated that SERS technique with GNRs can detect the limit of detection for 0.25 mg/L of chlormequat chloride in wheat extraction solution.

### 3.2. Spectral Analysis Using Chemometric Methods

**Figure 5:**
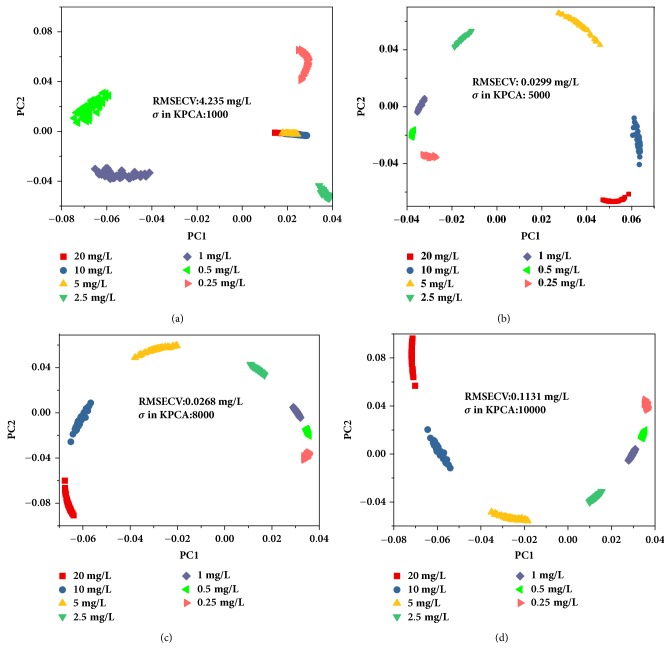
Scatter plot of first two principal component scores obtained by KPCA with *σ* of 1000 (a), 5000 (b), 8000 (c), and 10000 (d); PC1: the first principal component. PC2: the second principal component.

**Figure 6:**
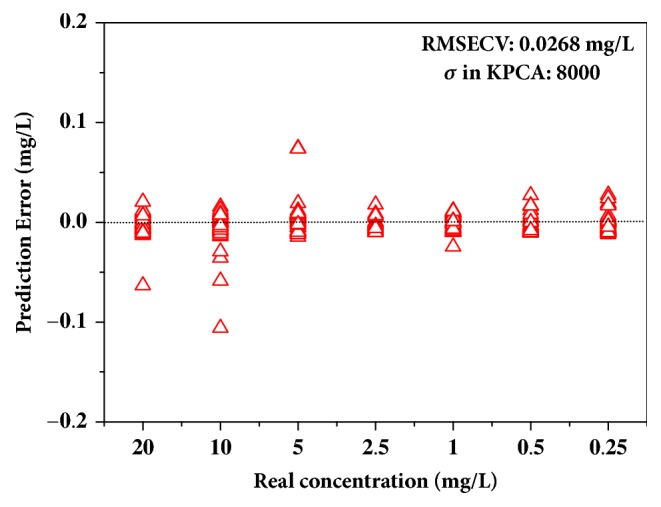
Predicted error of the optimal model built using SVR and KPCA with *σ* of 8000.

Intelligent analysis of spectra using chemometric methods can automatically obtain the information of substances without intervention of professionals, and this process is of significance for simple and rapid detection. SERS spectra are of high dimension and carry useless information for target analyte. The appropriate variable selection and feature extraction can improve the analysis results. Considering the fingerprint properties of SERS, the spectra around characteristic peaks were selected for the intelligent analysis, and the interference can be avoided from the irrelevant information in spectra of other ranges. In particular, for SERS of chlormequat chloride in wheat extraction, the spectra of 653–683, 705–728, and 847–872 cm^−1^ were selected for the subsequent analysis. Then, KPCA with RBF was adopted to extract the principal feature of processed spectra. Feature extraction was highly dependent on *σ* in RBF, and the effects of different *σ* were discussed.  Figure 5 shows the scatter of first two principal component scores obtained by KPCA with *σ* of 1000, 5000, 8000, and 10000. When *σ* was 1000, the corresponding score of wheat extraction with 20, 10, and 5 mg/L chlormequat chloride overlapped one another, which suggests bad subsequent results. However, for other larger values, the scatter of each category was separated well. Afterward, the obtained first two principal component scores were employed to develop the regression models for quantitative determination, and the model performance was evaluated with RMSECV (Table 1). As can be seen from Table 1, the RMSECV value of the linear model established with MLR and PLSR is relatively large, which may lead to the inaccurate prediction results. Meanwhile, the model obtained by SVR and KPCA with *σ* of 1000 was worse than others and consistent with the above assumption. Too large *σ* (10000) also caused the quantitative analysis results to yield poor results [38]. Accordingly, SVR and KPCA with *σ* of 8000 was used for quantitative determination of chlormequat chloride, and the predicted error of the optimal model is shown in Figure 6. From the figure, the concentration of all samples was accurately predicted with small error, and RMSECV of the model was 0.0268 mg/L. In conclusion, SERS and SVR with KPCA can provide an accurate detection method for chlormequat chloride residue in wheat solution. Subsequently, the optimal established model would be used to predict concentration of residue in the real case.

**Figure 7:**
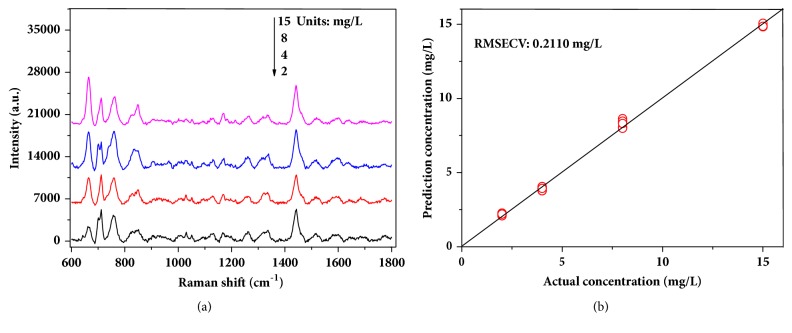
Spectra of 15, 8, 4, and 2 mg/L of chlormequat chloride in wheat extraction solution (a), predicted results by using SVR and KPCA (b).

In addition, an unbiased estimation for generalization of the model was conducted with an independent testing set. The independent testing set was spectra of wheat extraction with 15, 8, 4, and 2 mg/L obtained through remeasurement, and the representative spectra were shown in Figure 7(a). From figure it is known that the representative spectra were almost identical to the previously measured spectra. The predicted error (RMSECV) for the new testing set with the optimal model was about 0.2110 mg/L (Figure 7(b)). Meanwhile, standard deviation was from 0.052 mg/L to 0.102 mg/L, and the predicted recovery was 97.4 %–110.3 % in Table 2. The above results indicated that concentration of residue can be accurately predicted, and the model established by SVR and KPCA had good generality.

### 3.3. Quantification of Chlormequat Chloride Residue in Wheat Samples

**Figure 8:**
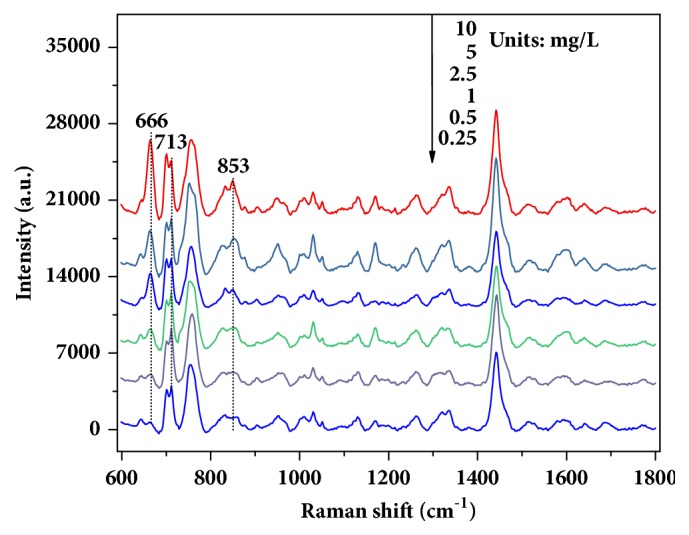
Spectra of wheat samples spiked with chlormequat chloride at 10, 5, 2.5, 1, 0.5, or 0.25 *μ*g/g.

To simulate actual residue, wheat powder was spiked with chlormequat chloride to yield final residue at 10, 5, 2.5, 1, 0.5, and 0.25 *μ*g/g. The contaminated samples were extracted using the two-step procedure, and the obtained extraction solution was directly used for SERS measurement. For the residue of each concentration, 50 spectra were collected from five samples, respectively. The representative spectra are shown in Figure 8. From figure, spectra of chlormequat chloride residue in wheat were highly consistent with the spectra of residue in extraction solution, and the characteristic peaks at 713, 666, and 853 cm^−1^ were still obvious and feasible for quantification of analyte. However, a small difference in spectral intensity can be observed, which depended on the extraction efficiency of pretreatment methods for residue in wheat samples.

Afterward, all the spectra were processed using KPCA, and the first two principal component scores were used to predict the sample concentration combining with the established model. The experiment results are shown in Table 3. The predicted recovery was in the range of 94.7 % to 104.6 %, and standard deviation was from 0.007 mg/L to 0.066 mg/L. Results proved that the proposed pretreatment method was feasible and effective for extraction of chlormequat chloride residue in wheat. In addition, the lowest tested concentration of 0.25 *μ*g/g was far below maximum residue limit of chlormequat chloride in wheat (5 *μ*g/g). These results also demonstrated SERS and SVR with KPCA can realize accurate quantification of residue with good repeatability and high sensitivity. Furthermore, the portable Raman spectrometer made the quantitative determination easy and efficient to be performed. In the future, the presented method can be applied for the detection of various farm chemicals in other grains.

## 4. Conclusions

In this work, a method for detection of chlormequat chloride in wheat was developed using SERS and chemometric methods on a portable Raman spectrometer. The extraction of residue in wheat was performed using a two-step procedure originated from GC detection. As for the spiked wheat samples, the optimal predicted recovery was in the range of 94.7 % to 104.6 %, and standard deviation was from 0.007 mg/L to 0.066 mg/L. These results indicated that the present method is an effective and feasible approach for determination of chlormequat chloride residue in wheat. Meanwhile, with aid of a portable Raman spectrometer, the present method could be executed onsite, which is suitable for rapid residue analysis in grains. However, spectral variation induced by instability of substrate and differences in sample pretreatment should be avoided and resolved prior to application of SERS. In conclusion, SERS with chemometric methods is a potentially powerful approach for detecting chlormequat chloride or other toxic residues in grains which can greatly help improve the safety and quality of agricultural products.

## Figures and Tables

**Table 1:** Predicted results of the model developed using chemometric methods.

Data	MLR	PLSR	KPCA+SVR	
	RMSECV (mg/L)	RMSECV (mg/L)	*σ* in KPCA	RMSECV (mg/L)
Spectra of 653-683, 705-728, and 847-872 cm^−1^	0.3757	0.3758	1000	4.235
			5000	0.0299
			8000	0.0268
			10000	0.1131

**Table 2:** Predicted results of 15, 8, 4, and 2 mg/L of chlormequat chloride in wheat extraction solution using SERS, SVR, and KPCA.

Spiked value (*μ*g/g)	Mean predicted value (mg/L)	Standard deviation (mg/L)	Recovery (%)
15	14.87	0.066	99.1
8	8.18	0.091	102.3
4	3.90	0.052	97.4
2	2.21	0.102	110.3

**Table 3:** Predicted results of chlormequat chloride in wheat using SERS, SVR, and KPCA.

Spiked value (*μ*g/g)	Mean predicted value (mg/L)	Standard deviation (mg/L)	Recovery (%)
10	9.96	0.066	99.6
5	4.74	0.042	94.7
2.5	2.614	0.064	104.6
1	1.012	0.014	101.2
0.5	0.479	0.007	95.8
0.25	0.242	0.025	96.8

## Data Availability

The data used in the article can be downloaded and viewed at the following address: https://pan.baidu.com/s/1jJr4jvo.
